# Supplementary material for: Long-term consequences of the absence of leptin signaling in early life
Source: eLife. 2019 Jan 29;8:e40970. doi: 10.7554/eLife.40970 (PMC6384028; doi:10.7554/eLife.40970)
Supplement: Figure 3—source data 2. [file elife-40970-fig3-data2.docx]

Primer sequences for quantitative PCR.

| **Gene** | **Primer Forward (5'-3')** | **Primer Reverse (3'-5')** |
| --- | --- | --- |
| ***Agrp*** | ctttggcggaggtgctagat | aggactcgtgcagccttacac |
| ***Actb*** | catcgtgggccgctcta | cacccacataggagtccttctg |
| ***Bdnf*** | gccttcatgcaaccgaagta | atgttccaccaggtgagaag |
| ***Cartpt*** | cagtcacacagcttcccgat | cagatcgaagcgttgcaaga |
| ***Dlg4*** | cccagacctgagttacccctt | agttgcaggtgaacggaaca |
| ***Dnmt1*** | ccggaaactcacttggacga | tttggcagctggatctctgg |
| ***Dnmt3a*** | attgatgagcgcacaaggga | gtgacattgaggctcccaca |
| ***Dnmt3b*** | tcctggcatgtaacccagt | tctttgaagccatcacgggc |
| ***Gapdh*** | cggcagcccagaacatcat | ccgttcagctctgggatga |
| ***Gfap*** | gctagccctggacatcgaga | ccccttctttggtgcttttgc |
| ***Hdac3*** | ggcctggtaaggcttgaaga | ggagctggacacccaatgaa |
| ***Hdac5*** | ctccagtgtttgctctggatctc | gcagcccttggatgaagcag |
| ***Hdac8*** | agttctggtgaaacaggctct | ctgatgttggcctggggaaa |
| ***Igf1*** | gtacttcctttccttctcctttgc | ccacactgacatgcccaaga |
| ***Kiss1*** | gattccttttcccaggcatt | ggcaaaagtgaagcctggat |
| ***Lepr (b)*** | tgtcctactgctcggaacac | gctcaaatgtttcaggcttttgg |
| ***Nos1*** | cggaccttgtagctcttcctc | ttcggctgtgctttgatgga |
| ***Npy*** | cagatactactccgctctgcg | gggctggatctcttgccata |
| ***Pomc*** | tagatgtgtggagctggtgc | ccagcgagaggtcgagtttg |
| ***Ppia*** | cttcttgctggtcttgccattcc | tatctgcactgccaagactgagt |
| ***Prlh*** | ctgctgctaggcttagtcctc | ggattgatgtcaggggtgcg |
